# 基于场放大进样和石墨烯量子点双重富集毛细管电泳分离检测三聚氰胺和双氰胺

**DOI:** 10.3724/SP.J.1123.2021.08017

**Published:** 2022-03-08

**Authors:** Chao LI, Qi WANG, Zhaoxiang ZHANG

**Affiliations:** 光电传感与生命分析教育部重点实验室, 青岛科技大学化学与分子工程学院, 山东 青岛 266042; College of Chemistry and Molecular Engineering, Qingdao University of Science and Technology, Qingdao 266042, China; 光电传感与生命分析教育部重点实验室, 青岛科技大学化学与分子工程学院, 山东 青岛 266042; College of Chemistry and Molecular Engineering, Qingdao University of Science and Technology, Qingdao 266042, China; 光电传感与生命分析教育部重点实验室, 青岛科技大学化学与分子工程学院, 山东 青岛 266042; College of Chemistry and Molecular Engineering, Qingdao University of Science and Technology, Qingdao 266042, China

**Keywords:** 硫掺杂石墨烯量子点, 毛细管电泳, 三聚氰胺, 双氰胺, sulfur-doped graphene quantum dots (S-GQDs), capillary electrophoresis (CE), melamine, dicyandiamide

## Abstract

通过热解法制备了硫掺杂的石墨烯量子点(S-GQDs),同石墨烯量子点(GQDs)相比,S原子的引入有效改善了GQDs的表面状态和化学特性、增强其对正电荷的捕获能力,使其更易与阳离子相互作用。以S-GQDs为载体,结合电堆积富集技术,发展了一种基于场放大进样(FASI)和S-GQDs放大的双重富集毛细管电泳(CE)分离检测三聚氰胺和双氰胺的方法。三聚氰胺和双氰胺在酸性介质中带正电荷,电动进样时快速迁移到毛细管入口端进行FASI预富集;同时带负电荷的S-GQDs向阳极端迁移,在样品与缓冲溶液的界面处通过静电作用吸附样品离子,S-GQDs作为载体使检测信号进一步放大。实验考察了缓冲溶液中S-GQDs的体积分数、缓冲溶液的组成及pH、进样时间等因素对富集分离效果的影响。当缓冲溶液的pH为4.6时,进样时间可延长至450 s。同常规电动进样(10 kV×10 s)相比,采用FASI与S-GQDs双重放大技术可使检测灵敏度提高1.6×10^5^倍。该方法对三聚氰胺和双氰胺检测的线性范围是1.0×10^-14^~1.0×10^-8^mol/L,相关系数(*r*^2^)大于0.999,检出限分别为2.6×10^-15^和5.7×10^-15^mol/L。实现了对盐酸二甲双胍中三聚氰胺和双氰胺的高灵敏检测,回收率分别为95.9%~102.4%和92.0%~106.0%,相对标准偏差(RSD)小于5%。该方法操作简单,分离效果好,准确度高,重现性好,适用于分离检测不同盐酸二甲双胍制剂中的三聚氰胺和双氰胺。

## Abstract (en)

Sulfur-doped graphene quantum dots (S-GQDs) were prepared by the pyrolysis of citric acid and mercaptopropionic acid. Compared with graphene quantum dots (GQDs), the S-GQDs have improved surface state and chemical reactivity, and thus, exhibited stronger interaction with cations. Based on its excellent affinity for cations, a dual preconcentration technique combining field-amplified sample injection (FASI) and S-GQDs as multianalyte carriers was developed for the determination of melamine and dicyandiamide by capillary electrophoresis (CE). During the FASI process, a large quantity of analytes was introduced into the capillary and accumulated at the capillary inlet. Concurrently, the S-GQDs migrated to the anode and captured the analytes on its surface at the boundary of the sample and buffer solution. The use of S-GQDs allows the capture of abundant analytes, which can amplify the detection signal. This new protocol was evaluated by the quantitative determination of melamine and dicyandiamide in metformin hydrochloride preparations. The effect of volume fraction of the S-GQDs in the buffer solution, the composition and pH of the buffer, and the sample injection time on the preconcentration and separation were investigated. By controlling the pH at 4.6, the sample injection time was prolonged to 450 s. A very large amount of melamine and dicyandiamide, bearing positive electric charges, were injected into the capillary and were captured by S-GQDs. The assay using FASI preconcentration and S-GQDs as enhancer resulted in a 1.6×10^5^-fold improved sensitivity compared with that obtained with traditional 10-kV electrokinetic injection for 10 s. The calibration curves of melamine and dicyandiamide were obtained in the concentration range from 1.0×10^-14^ to 1.0×10^-8^mol/L, with correlation coefficients (*r*^2^) >0.999. The detection limits (*S/N*=3) were 2.6×10^-15^mol/L for melamine and 5.7×10^-15^mol/L for dicyandiamide. The recoveries of the two analytes were 95.9%-102.4% and 92.0%-106.0%, respectively, with relative standard deviations (RSDs) of no more than 5%. The RSD values of peak height, peak area, and migration time were all less than 5.6%. This method is reliable, easy, and exhibits a good separation effect. This proves that the S-GQD-enhanced CE method could be developed into a new and sensitive technique for the determination of melamine and dicyandiamide in different preparations of metformin hydrochloride.

二甲双胍为口服类降血糖药,主要用于治疗II型糖尿病。三聚氰胺和双氰胺是合成二甲双胍的中间体和前驱体,在二甲双胍合成工艺中,不可避免地会引入三聚氰胺和双氰胺,二者毒性较大,必须严格控制其在药物中的含量。目前检测三聚氰胺和双氰胺的方法主要有液相色谱-质谱法^[1,2,3]^、磁性表面分子印迹-色谱法^[4]^、毛细管电泳法(CE)^[5]^等。CE以其分离效率高、样品用量少等特点,在复杂样品的分离检测中有着不可替代的优势,但CE-紫外(UV)检测的灵敏度受待测物浓度的限制。通过在线浓缩富集技术可以提高检测灵敏度^[6,7,8]^。

近年来,随着纳米技术的迅速发展,磁性纳米材料^[9,10]^、金纳米粒子^[11,12,13]^等作为标记、富集或分离介质,已广泛用于分析检测中。石墨烯量子点(graphene quantum dots, GQDs)是一类有独特光电子性能的纳米材料,具有优良的荧光特性,且有毒性低、易于化学修饰等特点,常被用于传感器、生物成像、光催化等领域^[14,15,16,17]^。Li等^[18]^合成了硫掺杂的GQDs作为荧光探针高灵敏检测Fe^3+^。Chen等^[19]^通过切割氮掺杂氧化石墨烯合成了具有双电位电化学发光响应的氮掺杂GQDs,并通过对比不同激发电位下电化学发光强度的比值实现对Co^2+^的定量检测。Chen等^[20]^通过电解法合成了硼掺杂的GQDs作为荧光传感器高灵敏检测水中的Fe^3+^。Anh等^[21]^合成了尺寸可调的金硫掺杂GQDs高灵敏检测硝基苯酚,检出限达8.4 nmol/L。掺杂型GQDs富含多种官能团,水溶性好,比表面积大,可以与多种物质相互作用,但迄今为止,掺杂型GQDs主要用于光学分析,对其负载能力的研究还未见文献报道。

本文利用硫掺杂石墨烯量子点(sulfur-doped graphene quantum dots, S-GQDs)做载体,结合电堆积富集技术,发展了一种基于场放大进样(field-amplified sample injection, FASI)和S-GQDs放大的双重富集CE分离检测三聚氰胺和双氰胺。S-GQDs表面富含羟基、羧基和磺酸基,通过静电作用对正电荷有很强的捕获能力,结合FASI技术,使检测信号得到极大的提高。

## 1 实验部分

### 1.1 仪器与试剂

HP毛细管电泳仪(Agilent公司);熔融石英毛细管(内径50 μm,外径365 μm,河北永年锐沣色谱器件有限公司); H7100透射电子显微镜(日本Hitachi公司);紫外可见光谱仪(美国Perkin Elmer公司); FT-IR 6600傅里叶变换红外光谱仪(江苏天瑞仪器有限公司); pHSJ-4A型酸度计(上海精密科学仪器有限公司)。

三聚氰胺和双氰胺标准品(日本东京化成工业株式会社);盐酸二甲双胍(山东齐都药业有限公司);柠檬酸、3-巯基丙酸(阿拉丁试剂有限公司);磷酸二氢钠、NaOH、HCl(中国医药集团上海化学试剂公司)。所用试剂均为分析纯,实验用水为去离子水。

### 1.2 标准溶液的配制

0.01 mol/L三聚氰胺和双氰胺储备液:分别称取31.5 mg三聚氰胺和21.0 mg双氰胺,加入5 mL甲醇溶解,然后加入去离子水使其完全溶解后转入25 mL容量瓶中,定容,摇匀。使用时用50 mmol/L pH 4.6的磷酸盐缓冲溶液稀释至所需浓度。

### 1.3 S-GQDs的制备

如图1a所示,通过热解法制备S-GQDs^[21]^:将2.0 g柠檬酸和0.3 mL 3 mmol/L 3-巯基丙酸置于5 mL四氟乙烯为内衬的不锈钢反应釜中,密封后置于烘箱,于200 ℃反应45 min,取上清液用1000 Da的透析袋纯化处理1天,即得纯化的S-GQDs。

**图1:**
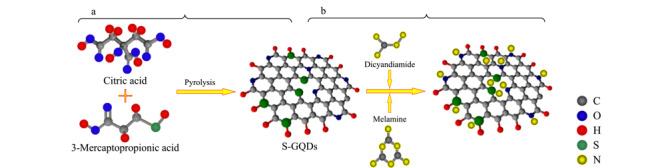
(a)S-GQDs的制备和(b)三聚氰胺和双氰胺在S-GQDs上的吸附

### 1.4 CE分离条件及检测原理

基于FASI和S-GQDs的双重富集CE分离检测原理如图2和图1b所示,毛细管内充满50 mmol/L pH 4.6的磷酸盐缓冲溶液(含25%的S-GQDs), 10 kV电动进样450 s,带正电荷的三聚氰胺和双氰胺快速向毛细管迁移,并在毛细管入口端进行FASI预富集(见图2a);同时缓冲溶液中带负电荷的S-GQDs向阳极迁移,由于S-GQDs表面存在大量的羧基和磺酸基,在毛细管入口端通过静电作用吸附样品离子(见图2b和图1b),由于S-GQDs的表面积/体积比大,其吸附容量很高,能将大量样品粒子吸附到其表面,低电渗流和S-GQDs的高吸附容量使进样时间达450 s,检测信号得到极大程度的放大;吸附了样品离子的S-GQDs表面带上大量正电荷,使其电泳淌度转向阴极端,在电泳和电渗流的共同作用下,S-GQDs载带着样品离子向阴极迁移,进行分离检测(见图2c),检测波长210 nm。

**图2:**
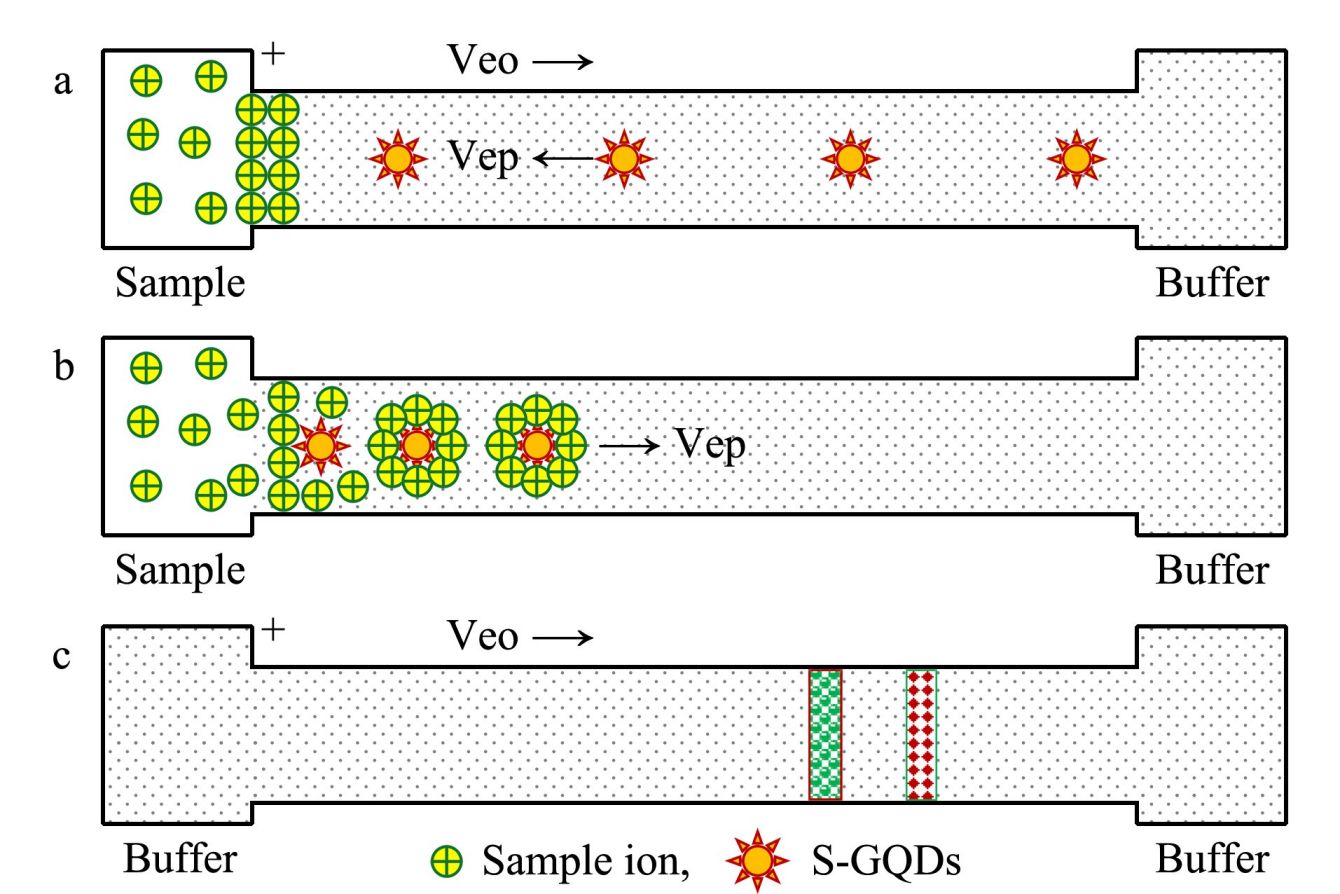
基于场放大进样和石墨烯量子点双重富集的CE分离检测示意图

### 1.5 样品处理

将二甲双胍药片研磨,过60目(0.25 mm)筛,称取0.05 g药品粉末置于10 mL具塞塑料管中,加入5 mL乙醇溶解,超声30 min后,13000 r/min离心10 min,取上清液,置于100 mL容量瓶中,定容,备用。

## 2 结果与讨论

### 2.1 S-GQDs的表征

S-GQDs的透射电镜(TEM)图(见图3a)和粒径分布图(见图3b)表明,所制备的S-GQDs呈球形,分散度好,粒径均匀,平均粒径4.7 nm; X射线衍射谱(XRD)图(见图3c)中2*θ*在23.3°附近的衍射峰对应于石墨烯结构^[22]^。

**图3:**
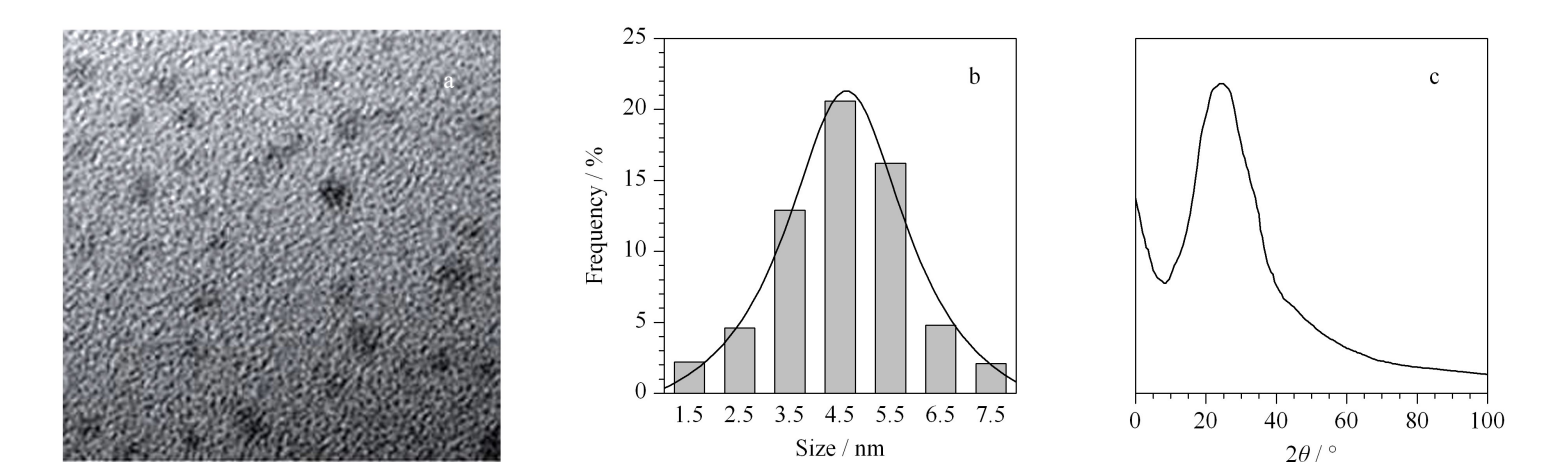
S-GQDs的(a)TEM图、(b)粒径分布图和(c)XRD谱图

图4a为S-GQDs的X射线光电子能谱(XPS)图,在160、225、290和540 eV处观测到4个初级峰,分别对应于S 2*p*、S 2*s*、C 1*s*和O 1*s*, S 2*s*峰和S 2*p*峰表明硫原子成功掺杂到GQDs中。图4b为S 2*p*的高分辨XPS谱,162.4 eV的峰值表明存在C-S(O_2_)-C单元。对XPS能谱分析可得S-GQDs中各元素含量百分比,S元素的含量达到16.25%,说明该制备方法能高效掺杂硫。XPS结果表明,S-GQDs富含磺酸基、羧基和羰基等官能团,这不仅使S-GQDs水溶性好,也为其作为载体通过静电作用吸附阳离子提供可能。

**图4:**
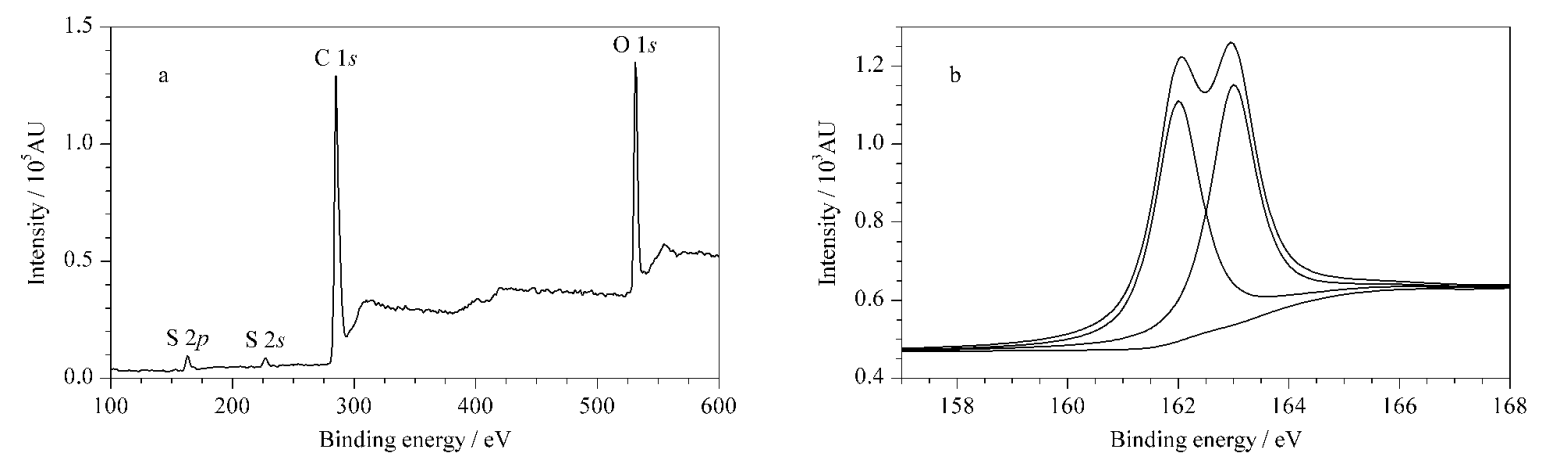
(a) S-GQDs的XPS全谱图和(b)S 2*p*的高分辨XPS谱图

### 2.2 S-GQDs富集

S-GQDs比表面积大,含有大量的羧基、磺酸基等官能团,可通过静电作用与阳离子结合,是阳离子的优良载体,本文将制备的S-GQDs加入到缓冲溶液中,使大量待测离子吸附到S-GQDs表面,对检测信号起到极大的放大作用。

将不同体积的S-GQDs加入到缓冲溶液中,使S-GQDs所占的体积分数为0%~50%,如图5所示,随着S-GQDs所占体积分数增大,三聚氰胺和双氰胺的峰高、分离度和分离效率(*N*=16× $t_{R}^{2}$ /*W*, *N*为理论塔板数;*t*_R_为保留时间;*W*为峰宽)都增大,说明S-GQDs可以作为载体吸附三聚氰胺和双氰胺。然而,当S-GQDs的体积分数大于30%时,三聚氰胺和双氰胺的分离效率减小,峰高和迁移时间的重现性变差,这可能是由于S-GQDs在缓冲溶液中的体积分数太高影响了后续的分离,同时考虑富集和分离的效果,缓冲溶液中S-GQDs的体积分数选择为25%。

**图5:**
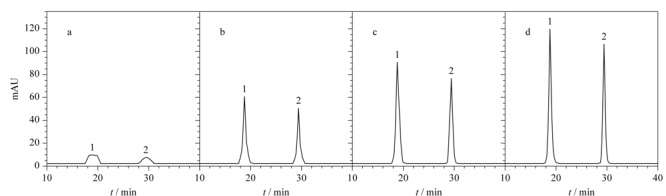
缓冲溶液中S-GQDs的体积分数对三聚氰胺和双氰胺富集分离的影响

### 2.3 缓冲溶液的选择

三聚氰胺和双氰胺都是碱性化合物,在pH小于8时带正电荷。研究了不同浓度和pH的磷酸盐、醋酸盐、柠檬酸盐等缓冲溶液对分离三聚氰胺和双氰胺的影响,结果发现,用磷酸盐缓冲溶液分离效果最好。改变磷酸盐缓冲溶液的浓度(10~80 mmol/L),发现在50 mmol/L时三聚氰胺和双氰胺的分离度最大,分离效率最高。

缓冲溶液的pH对三聚氰胺和双氰胺的富集和分离效果影响显著。电动进样时,带正电荷的样品离子与S-GQDs迎面相遇,由于静电作用被吸附到S-GQDs表面,使S-GQDs表面带上正电荷;吸附的三聚氰胺和双氰胺离子越多,S-GQDs表面的正电荷就越多,从而导致S-GQDs向阳极方向的迁移速率减小,甚至停止或转向。如果富集界面在进样过程中快速向检测端迁移,为留出足够长的毛细管进行后续分离,进样时间就受到限制。S-GQDs的迁移速率(*V*_S-GQDs_)与其电泳速率(*V*_ep,S-GQDs_)及电渗流速率(*V*_eo_)的关系为:


(1) *V*_S-GQDs_=*V*_eo_+*V*_ep,S-GQDs_

在界面处S-GQDs与三聚氰胺和双氰胺作用后,形成的复合粒子的迁移速率(*V*_R-N-S-GQDs_)可表示为:


(2) *V*_R-N-S-GQDs_=*V*_eo_+*V*_ep,R-N-S-GQDs_

从式(2)可以看出,减小电渗流速率*V*_eo_,可使复合粒子的迁移速率减小,允许较长的进样时间,增大检测灵敏度。研究了缓冲溶液的pH(3.0~7.0)对检测灵敏度、分离效率及迁移时间的影响,结果发现,pH在3.0~4.6范围内,可以允许较长的进样时间,检测灵敏度高,且随着pH的增大,迁移时间减小,分离效率增大;当pH在4.6~7.0范围内时,随着pH的增大,允许的进样时间缩短,检测灵敏度降低。综合考虑灵敏度和分离效率,选择缓冲溶液的pH为4.6。

### 2.4 进样时间的选择

S-GQDs的吸附容量限制了FASI时间,如图6所示,随着进样时间的增大,三聚氰胺和双氰胺峰高增大,当进样时间超过450 s后,峰高增大的趋势变小,分离效率降低。同时,在电泳谱图中发现,当进样时间为450 s时,从进样开始到分离检测结束,未检测到游离的三聚氰胺和双氰胺,说明450 s的进样时间引入的三聚氰胺和双氰胺峰未超出S-GQDs的吸附容量,进入毛细管的三聚氰胺和双氰胺经FASI预富集后全部被S-GQDs吸附,有效增大了检测灵敏度,因此,选择进样时间为450 s。

**图6:**
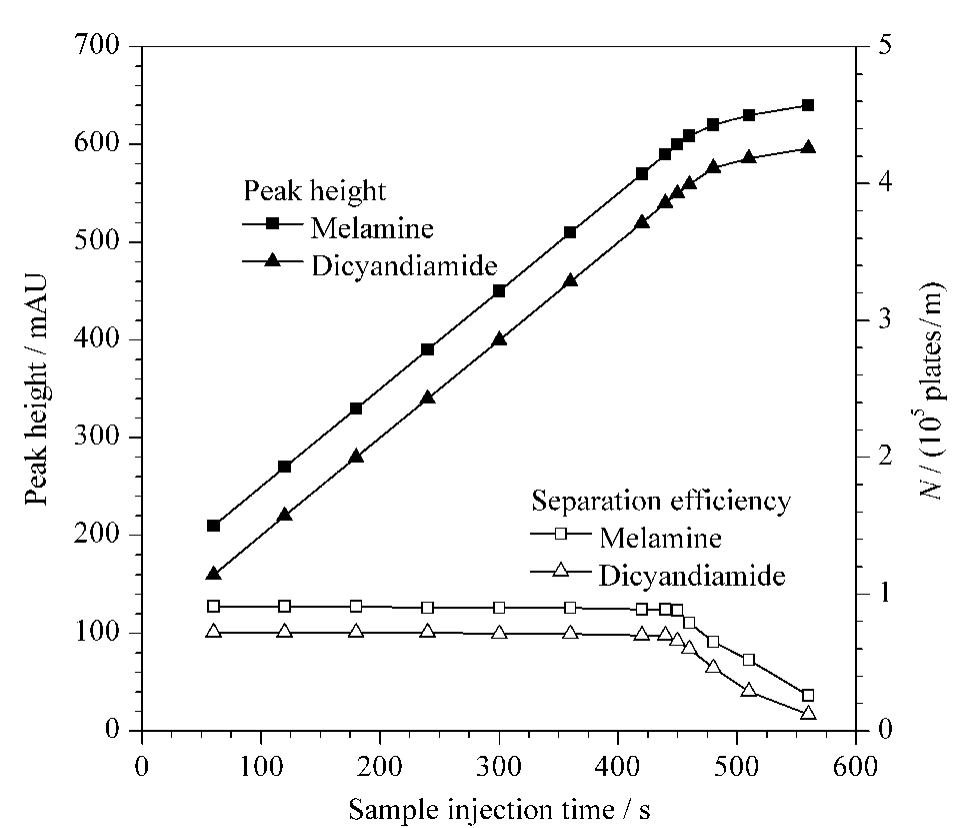
进样时间对三聚氰胺和双氰胺的峰高和分离效率的影响

由于FASI过程中富集界面向检测端的迁移速率很慢,因此可使进样时间达到450 s而不影响后续分离;同时,堆积在界面上的样品离子与迎面而来的S-GQDs相互作用后,其向检测端的迁移速率很慢,样品离子与S-GQDs有足够的时间相互作用,使样品离子在S-GQDs表面得到最大程度的吸附,检测信号得到极大程度的提高。如图7所示,同常规电动进样(10 kV×10 s)相比,采用FASI与S-GQDs双重放大技术可使检测灵敏度提高1.6×10^5^倍(峰高比×样品稀释因子)。

**图7:**
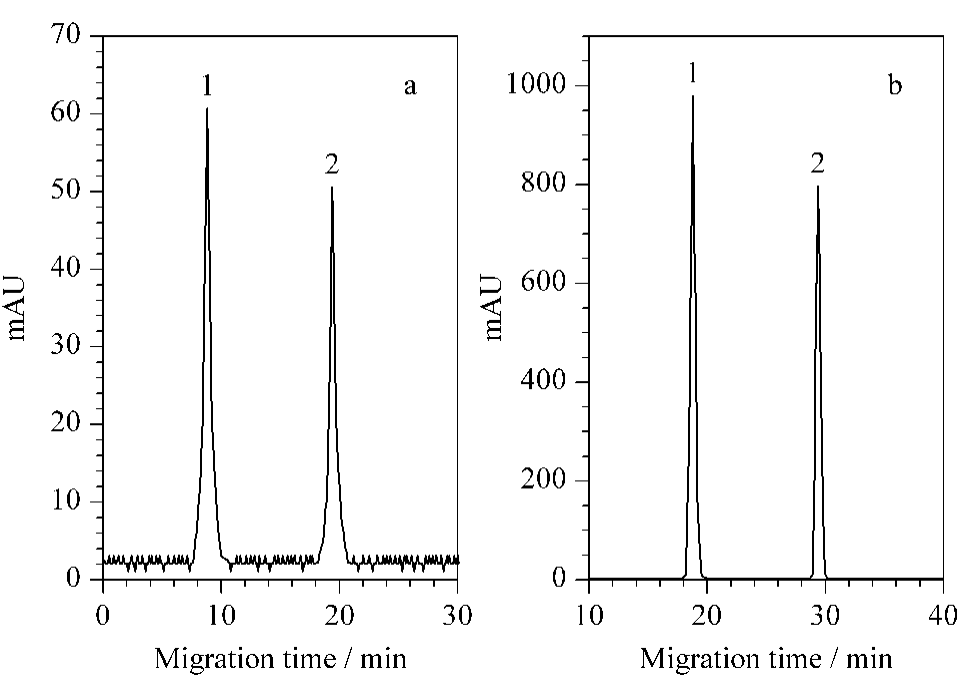
富集前后对比

### 2.5 方法学考察

在50 mmol/L pH 4.6的磷酸盐缓冲溶液(含25%的S-GQDs)、10 kV电动进样450 s条件下,研究了三聚氰胺和双氰胺的工作曲线、线性范围、检出限和精密度(相对标准偏差,RSD),如表1所示。以三聚氰胺和双氰胺的浓度(*c*, mol/L)的负对数(*x*=-log *c*)为横坐标,对应的峰高(*y*)为纵坐标绘制工作曲线,结果表明,两种化合物在1.0×10^-14^~1.0×10^-8^mol/L范围内呈现良好的线性关系,相关系数(*r*^2^)大于0.999,浓度检出限(LOD, *S/N*=3)分别为2.6×10^-15^和5.7×10^-15^mol/L。

**表1:** 三聚氰胺和双氰胺的回归方程、相关系数、线性范围、检出限和精密度(*n*=5)

Compound	Regression equation	r^2^	linear range/ (mol/L)	LOD/ (mol/L)	RSDs/%		
					Peak height	Peak area	Migration time
Melamine	y=-238.8x+3349.1	0.9996	10^-14^-10^-8^	2.6×10^-15^	2.8	2.6	4.3
Dicyandiamide	y=-219.6x+3074.3	0.9992	10^-14^-10^-8^	5.7×10^-15^	3.2	3.7	5.6
y: peak height; x: -log c; c: concentration, mol/L.							

比较了文献报道的不同方法检测三聚氰胺和双氰胺的检出限,如表2所示,通过表中数据可以看出,本文方法具有最低的检出限。

**表2:** 与其他测定三聚氰胺和双氰胺的方法的比较

Method	Detection limits				Reference
	Melamine		Dicyandiamide		
LC-MS	54	μg/L	5.4	μg/L	[1]
HPLC-MS	0.1	mg/kg	0.4	mg/kg	[2]
Tandem dual solid phase extraction cartridges-HPLC-electrospray	1.48	μg/kg	13.61	μg/kg	[3]
ionization multi-stage MS					
Magnetic surface molecularly imprinted polymers-HPLC	15	μg/L	-		[4]
FASI+S-GQDs CE	2.6×10^-15^	mol/L	5.7×10^-15^	mol/L	this work

-: undetected.

### 2.6 实际样品检测

将三聚氰胺和双氰胺的混合标准溶液加入二甲双胍样品中,按1.5节将样品处理好后进行回收率测定,如表3所示,三聚氰胺和双氰胺的回收率分别为95.9%~102.4%和92.0%~106.0%,其RSD均小于5%。对每个加标样品进行精密度试验,日内连续测定6次,连续测量5天,日内和日间回收率的RSD小于12%,重复性良好。

**表3:** 盐酸二甲双胍样品中三聚氰胺和双氰胺的加标回收率及其RSD(*n*=5)

Sample	Melamine						Dicyandiamide				
	Original/ (μg/L)	Added/ (μg/L)	Found/ (μg/L)	Recovery/ %	RSD/ %		Original/ (μg/L)	Added/ (μg/L)	Found/ (μg/L)	Recovery/ %	RSD/ %
1^#^	82.5	80.0	159.2	95.9	3.3		5.6	5.0	10.2	92.0	2.2
2^#^	67.9	80.0	149.8	102.4	2.9		1.2	5.0	6.5	106.0	4.4
3^#^	96.2	80.0	175.1	98.6	4.8		2.9	5.0	7.6	94.0	2.8

## 3 结论

本文制备了易与阳离子结合的S-GQDs,以其为载体,结合FASI技术,发展了一种双重富集的CE分析技术同时分离检测三聚氰胺和双氰胺。通过控制缓冲溶液的pH,使进样时间延长至450 s,不仅增大了进样量,同时使样品离子与S-GQDs有充足的时间相互作用,使样品离子在S-GQDs表面得到最大程度的吸附。该双重富集技术使灵敏度提高1.6×10^5^倍,对三聚氰胺和双氰胺的检出限达2.6×10^-15^和5.7×10^-15^mol/L。该法成功用于二甲双胍样品中三聚氰胺和双氰胺的高灵敏检测,对实际样品中三聚氰胺和双氰胺的检测有良好的应用前景。
